# A 20-year population-based study on the epidemiology, clinical features, treatment, and outcome of nodular lymphocyte predominant Hodgkin lymphoma

**DOI:** 10.1007/s00277-015-2578-6

**Published:** 2016-01-05

**Authors:** L. Strobbe, L. L. F. G. Valke, I. J. Diets, M. van den Brand, K. Aben, J. M. M. Raemaekers, K. M. Hebeda, J. H. J. M. van Krieken

**Affiliations:** Department of Hematology, Radboud University Medical Center, Geert Grooteplein 8, 6525 GA Nijmegen, Postbus 9101, 6500 HB Nijmegen, The Netherlands; Department of Pathology, Radboud University Medical Center, Nijmegen, The Netherlands; Department of Registry and Research, Comprehensive Cancer Center, Utrecht, The Netherlands; Department of Hematology, Rijnstate Hospital, Arnhem, The Netherlands

**Keywords:** Chemotherapy, Epidemiology, Nodular lymphocyte predominant Hodgkin lymphoma, Outcome, Radiotherapy

## Abstract

Nodular lymphocyte predominant Hodgkin lymphoma (NLPHL) is a subtype of Hodgkin lymphoma characterized by a unique clinical and histological presentation. Because of the rare nature of this disease, few large-scale studies are available. We conducted a cohort study in which patients were identified in the Netherlands Cancer Registry in the Southeast of the Netherlands between 1990 and 2010. Of these patients, we collected all clinical characteristics and re-reviewed pathologic material to confirm NLPHL diagnosis. Seventy-three histologically confirmed cases of NLPHL were analyzed with a median follow-up of 65 months (range 4–257 months). Median age at diagnosis was 43 years (range 1–87); 84.9 % of the patients were male; B symptoms were present in 5.5 %; and stage I/II disease was most common (75.4 %). Patients were primarily treated with radiotherapy (50.7 %), chemotherapy (26 %), combined modality (radiotherapy and chemotherapy) (11 %), or surgical excision with careful watch-and-wait (12.3 %). Relapses occurred in seven patients (9.6 %) after a median of 26 months (21–74 months). Six patients (8.2 %) developed histologic transformation to large cell lymphoma. Five patients (6.8 %) died during follow-up due to progression of NLPHL (*n* = 1), histologic transformation (*n* = 2) and intercurrent deaths (*n* = 2). The estimated 10-year overall survival was 94.0 % and the 10-year progression-free survival 75.8 %. Our study confirms the distinct characteristics of NLPHL with a relatively good long-term prognosis. It may be possible to reduce treatment intensity in early stage NLPHL without affecting long-term outcome.

## Introduction

Nodular lymphocyte predominant Hodgkin lymphoma (NLPHL) is a rare subtype of Hodgkin lymphoma (HL) that accounts for approximately 5 % of all cases of HL [1]. It is distinguished from classical Hodgkin lymphoma (cHL) by clinical, histological, and immunophenotypic characteristics. Histologically, NLPHL is marked by the presence of lymphocyte predominant (LP) cells [2], previously known as lymphocytic and histiocytic (L and H) or “popcorn” cells, embedded in nodules consisting of B cells and other reactive cells (mainly reactive T cells) [3]. In contrast to cHL, Reed-Sternberg and Hodgkin (RSH) cells are rarely seen in NLPHL, and immunohistochemistry shows a different pattern on the malignant cells; RSH cells typically express CD15 and CD30, whereas LP cells lack expression of these markers, but express B cell markers like CD20, CD22, and CD79a and also express the common leukocyte antigen CD45, which is uncommon on RSH cells [4].

NLPHL was first recognized as a distinct disease entity in the revised European American lymphoma (REAL) classification of 1994, while beforehand NLPHL, was classified as cHL [5]. Hence, studies published before 1994 are often less representative for analysis of clinical presentation, treatment, and outcome. Furthermore, re-review of patient material on both morphological and immunohistochemical characteristics by an expert hematopathologist to confirm NLPHL is needed, as one study showed that only 51 % of originally diagnosed NLPHL cases was confirmed [1]. Because of the rare nature of this disease, few large-scale studies are available.

In comparison with cHL, NLPHL presents more often in an early stage of disease and has a male predominance (75 %) [1, 6–8]. In NLPHL, “B” symptoms are uncommon, as is bulky disease or involvement of mediastinal, abdominal, and extranodal sites [9]. The ideal treatment of NLPHL has not yet been established. Mostly, radiotherapy is used in case of limited disease (stage I and II), and combination therapy (chemotherapy and radiotherapy) is used in advanced-stage disease (like in cHL) [10]. Rituximab, an anti-CD20 antibody, is a promising therapy, but given the paucity of data, it is not yet considered first-line treatment of choice [11–13]. It is being increasingly recognized that late toxicities of treatment, like secondary malignancies and cardiovascular disease, can pose significant health problems and therefore treatment should be as limited as possible to prevent these late toxicities [14]. Furthermore, despite the good prognosis of NLPHL, recurrences are common, and there is a risk (of up to 13 %) of histologic transformation to non-Hodgkin lymphoma, typically diffuse large B cell lymphoma (DLBCL) [15].

The purpose of this study was to examine the epidemiology, clinical features, treatment, and outcome of patients with NLPHL, derived from the population-based cancer registry in the Southeast of the Netherlands, to get more insight in this rare subtype of Hodgkin lymphoma. Furthermore, we wanted to examine the percentage of discordant diagnoses through a central review of the histological material by expert lymphoma pathologists of our university hospital.

## Methods

All patients diagnosed with NLPHL in the Southeastern part of the Netherlands between 1990 and 2010 were identified through the Netherlands Cancer Registry (NCR). This region comprised 17 general hospitals and 1 university hospital. For all cases, histological material of the tumor was requested from the pathology labs and reviewed by an expert hematopathologist.

Standard data concerning patient and tumor characteristics were retrieved from the NCR. Retrospectively, additional data was collected by one researcher (I.D.) by consulting medical files using a standardized registration form. The following data were recorded: sex, age at time of diagnosis, presence or absence of B symptoms, Ann Arbor stage, presence of bulky mediastinal mass (tumor mass ≥10 cm; >0,35 mediastinum/thorax (MT) ratio), presence and number of involved lymph nodes, involvement of extranodal sites, the presence of European Organization for Research and Treatment of Cancer (EORTC) risk factors for stage I/II unfavorable risk disease [16], Hasenclever international prognostic score (IPS) for advanced-stage disease [17], presence of elevated lactate dehydrogenase (LDH), treatment, complications of treatment, date of recurrence, treatment of recurrence, histologic transformation, and date and cause of death.

Overall survival (OS) was defined as the time from date of diagnosis until the last date of follow-up or death, whichever came first. Progression-free survival (PFS) was measured from the date of diagnosis to the date of progression, transformation, or death from any cause, whichever occurred first. Early stage disease was defined as clinical stage I and II. Advanced-stage disease was defined as clinical stage III or IV.

Data were analyzed using Prism GraphPad software, version 5.0 (GraphPad Software, La Jolla, CA, USA). Standard descriptive statistical analyses were performed. Survival analysis was performed using the Kaplan-Meier method and presented as survival curves. Two-tailed *P* values <0.05 were considered statistically significant.

## Results

We identified 83 patients of whom three patients were excluded from further analysis because of unavailability of identification data. After pathology review, seven patients (8.8 %) were excluded because of discordant diagnosis; five patients were reclassified as cHL; and for two patients no conclusive diagnosis could be made. Thus, a total of 73 patients were included in the final analysis. The median follow-up time was 65 months (range 4–257 months).

The incidence of NLPHL increased during the study period from 6 new patients from 1990-1995 to 9 patients from 1996–2000, 20 patients from 2001–2005, and 38 patients in the most recent period (2006–2010). The incidence of NLPHL increased from 0.09 per 100,000 person years in 1990 to 0.30 per 100,000 person years in 2010, while the incidence of HL altogether remained stable during those years with an incidence of 2.31 per 100,000 person years in 1990 and 2.40 per 100,000 person years in 2010.

The main patient characteristics in comparison with recent studies are shown in Table 1. The median age of the patients was 43 years (range 1–87), and the majority of patients were male. We included eight children and they were diagnosed at the age of 1, 5, 9 (2), 12 (2), 14, and 15 years.

**Table 1:** Clinical characteristics of NLPHL patients in the current and recent studies

Characteristic	Our cohort	Farrell, 2011 [9]	Chen, 2010^a^ [20]	Biasoli, 2010 [18]	Al-Mansour, 2010 [15]
No. of patients	73	69	113	164	95
Follow-up (months; median and range)	65 (4–257)	53 (11–165)	136 (0–421)	114	78 (30–369)
Discordant diagnosis^b^ (%)	8.8	2.8	0	15.9	20
Male (number, %)	62 (85 %)	48 (70 %)	93 (82 %)	131 (80 %)	69 (73 %)
Age (median and range)	43 (1–87)	39 (11–79)	27 (3–77)	30 (8–69)	37 (15–77)
Stage (number, %)					
I	31 (43 %)	35 (51 %)	71 (63 %)	97 (60 %)	64 (67 %)^e^
II	24 (33 %)	21 (30 %)	42 (37 %)	60 (36 %)	^–^
III	12 (16 %)	9 (13 %)	0	6 (4 %)	31 (33 %)^f^
IV	5 (7 %)	4 (6 %)	0	0	^–^
Early stage disease	55 (75 %)	56 (81 %)	113 (100 %)	157 (96 %)	64 (67 %)
B symptoms^c^ (number, %)	4 (5.5 %)	3 (4.3 %)	0	7 (4.3 %)	8 (8.4 %)
Treatment modality (number, %)					
Watch-and-wait	31 (43 %)	5 (7 %)	0	58 (35 %)	2 (2 %)
Radiotherapy	37 (51 %)	37 (54 %)	93 (82 %)	43 (27 %)	26 (27 %)
Chemotherapy	19 (26 %)	15 (22 %)	7 (6 %)	15 (9 %)	31 (33 %)
Combine modality	8 (11 %)	12 (17 %)	13 (12 %)	48 (29 %)	33 (35 %)
Not specified	^–^	^–^	^–^	^–^	3 (3 %)
Deaths^d^ (number, %)	5 (6.8 %)	0	13 (11.5 %)	14 (8.5 %)	16 (16.8 %)
Transformation	6 (8.2 %)	2 (2.9 %)	NS	19 (11.2 %)	13 (14 %)

*NS* not specified

^a^This study only included early stage

^b^Percentage of excluded patients with a discordant diagnosis from the total number of patients that were reviewed

^c^B symptoms: fever, weight loss >10 % of body weight in 6 months and/or night sweats

^d^Deaths from all causes, not NLPHL-specific

^e^Stage I and II together

^f^Stage III and IV together

Early stage NLPHL was present in 75.4 % of the patients. B symptoms at the time of presentation were present in only 5.5 % of patients. In 55 patients with early stage disease, only nine had an unfavorable risk profile, and age was the only EORTC prognostic adverse risk factor for these patients. Patients with advanced disease scored at least 1 point in the Hasenclever score (median score of 2), with higher age and male sex as the most common risk factors.

One patient with stage II disease had extranodal disease of Waldeyers ring. None of the patients with stage III disease showed extranodal disease, but one patient had splenic involvement. In stage IV disease, splenic involvement was present in four of five patients, and four patients had skeletal involvement. Also, the thyroid gland, bone marrow, or liver was involved in one patient each.

Nine patients (12.3 %) had elevated LDH levels. Two of these patients died; three patients had a relapse; and four were cured without a relapse.

With regard to treatment, four different treatment options were used; a careful watch-and-wait strategy (after surgical excision of the only involved lymph node), involved-field radiotherapy, chemotherapy, or combined modality treatment (chemotherapy and radiotherapy). The different treatment modalities for different stages are shown in Table 2. Radiotherapy was the most used treatment option. Watch-and-wait was only used in nine patients with early stage disease; one of these nine patients refused treatment; one patient also had colorectal cancer for which treatment was given priority; five patients had long lasting lymphadenopathy (one patient already for 15 years); and two patients were children of 9 and 14 years old. Chemotherapy alone was mainly used in the advanced-stage group. Combined treatment modality was used in 11 % of the patients. Chemotherapy consisted of ABVD (adriamycin, bleomycin, vinblastine, dacarbazine) for a mean of 6 cycles (range 2–8) in eight patients, or MOPP-ABV (mechlorethamine, vincristine, procarbazine, prednisone-doxorubicin, bleomycin, vincristine) in six patients with a mean of 6 cycles (range 3–8). The combination of rituximab, cyclophosphamide, adriamycin, vincristine, and prednisone (R-CHOP) was used in four patients with a mean of 8 cycles, all in patients with localization in the spleen and/or stage IV disease, mainly because the advanced-stage presentation raised doubt on the representativity of the biopsied tissue for the diagnosis of NLPHL and the clinical suspicion of concordant presence of DLBCL. Other regimens included MOPP (in one patient, 6 cycles); vincristine, etoposide, doxorubicine, and prednisone (OEPA, in three pediatric patients, all 2 cycles); and cyclophosphamide, vincristine, prednisone (CVP) with rituximab (one patient, 3 cycles).

**Table 2:** First-line treatment of NLPHL patients according to stage

	Treatment			
Stage (no/%)	Watch-and-wait	Radiotherapy	Chemotherapy	Combined modality
I (31/42.5 %)	7 (22.6)	20 (64.5)	3 (9.6)	1 (3.2)
II (24/32.9 %)	2 (8.3)	15 (62.5)	4 (16.7)	3 (12.5)
III (12/16.4 %)	^–^	1 (8.3)	9 (75)	2 (16.7)
IV (5/6.8 %)	^–^	^–^	3 (60)	2 (40)
Unknown (1/1.4 %)	^–^	1 (100)	^–^	^–^
Total (73/100 %)	9 (12.3)	37 (50.7)	19 (26)	8 (11)

**Fig. 1:**
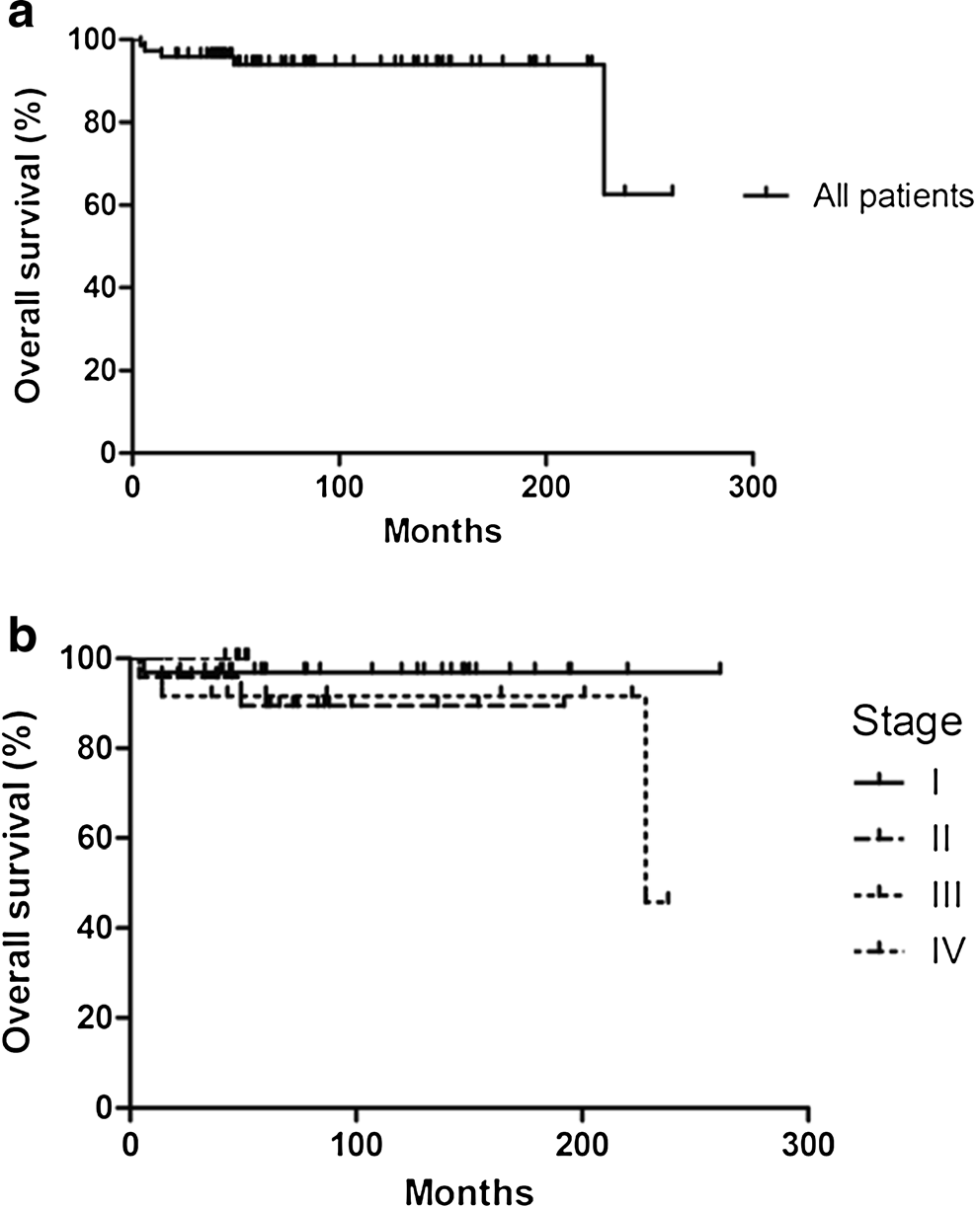
Overall survival in NLPHL patients is shown for all patients **(a)**, and broken-down according to stage **(b)**; stage I (*continuous line*), stage II (*long-dotted line*), stage III (*short-dotted line*), and stage IV (*long- and short-dotted line*)

Overall survival of patients with NLPHL is shown in Fig. 1. Two-year survival was 96.0 %; five-year survival was 94.0 %; and 10-year survival was 94 %. During follow-up, five patients (6.8 %) died. Three patients died as a result of NLPHL, and these all showed histologic transformation to DLBCL with transformation to a T cell-/histiocyte-rich large B cell lymphoma (T/HRBCL) in one of these patients. Two patients died of other causes; the cause of death was unknown for one patient, and the other patient died because of an infectious complication after lower anterior resection for colorectal cancer.

**Fig. 2:**
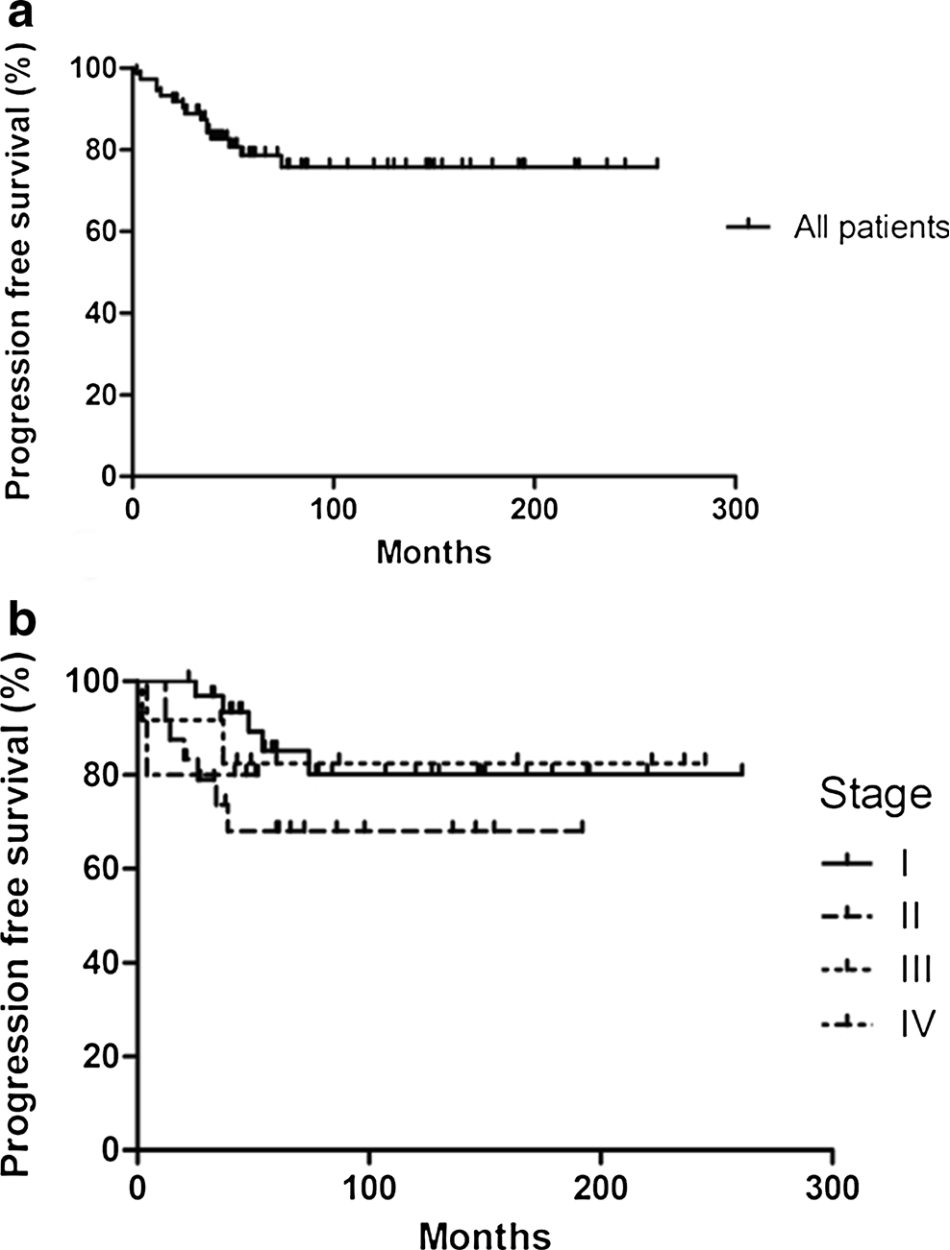
Progression-free survival in NLPHL patients is shown for all patients **(a)**, and broken-down according to stage **(b)**; stage I (*continuous line*), stage II (*long-dotted line*), stage III (*short-dotted line*) and stage IV (*long- and short-dotted line*)

Ten-year overall survival was 96.8 % for stage I disease, 89.4 % for stage II disease, 91.7 % for stage III disease, and 100 % for stage IV disease. Five-year OS was similar to 10-year OS. Progression-free survival was 78.6 % after 5 years and 75.8 % after 10 years (Fig. 2). Fig. 2b shows progression-free survival rates according to stage at presentation.

Six patients (8.2 %) had histologic transformation to DLBCL, two of which were of T/HRBCL subtype. These transformations all occurred in the first 5 years after diagnosis. Seven patients had a relapse of NLPHL after a median of 26 months (range 21–74 months). Relapses were mostly seen in patients with early stage disease, and patients who were treated with radiotherapy alone (*n* = 6) and in only one patient who was treated with chemotherapy (with 8 cycles of MOPP-ABV). None of the patients in the watch-and-wait group (with or without surgical excision) progressed.

## Discussion

In this population-based study, we examined the epidemiology, clinical features, treatment, and outcome of patients with NLPHL in the Southeastern part of the Netherlands. We confirmed the diagnosis of NLPHL by central expert hematopathological review, which caused exclusion of seven patients (8.8 %) because of a discordant diagnosis. This is in line with other studies which report discordance rates from 2.8 to 20 % [9, 15, 18]. As misclassification works in two directions, we cannot exclude that NLPHL patients have been classified as other malignant lymphomas (like cHL). We did not re-review histology of all patients with a diagnosis of malignant lymphoma over the same time period, so this could have influenced “true” incidence data.

During our study period, the incidence of NLPHL remarkably increased. This increase can be explained by several reasons. First, better recognition of NLPHL due to the definition of NLPHL as a distinct disease entity in the REAL classification in 1994 may have caused part of the increase, since incidence of total HL did not change during this period. Second, technical improvement in immunohistochemistry and availability of this technique could have contributed to the rise in incidence. Third, before 2000, recording of hematological malignancies from pathology or hematology labs possibly was not always accurate.

NLPHL is a unique disease entity with other clinical features than classic Hodgkin lymphoma [19]. Our study confirms the male predominance in NLPHL patients which was even more striking than in previous reports. In line with earlier studies, B symptoms were rare, and most patients presented in an early disease stage. The EORTC and IPS (Hasenclever) scores were recorded, but it has to be noted that these scores have only been validated for cHL and not for NLPHL.

Patients were treated with radiotherapy (50.7 %), chemotherapy (26 %), combined modality (11 %), or careful follow-up after removal of the lymph node (12.3 %). In other studies, all these treatment modalities were also used, but with different frequencies reflecting the lack of a standard treatment. Because of the small number of patients in subgroups, we were unable to perform statistical analyses on clinical markers, therapy, or outcomes.

We did not observe disease progression in any of the patients with a watch-and-wait approach (with or without surgical excision). Together with recent studies [18], this indicates that careful follow-up and treatment in case of progression can be a good option in patients with early stage disease who have only one affected lymph node that is completely removed by diagnostic biopsy. Several new treatment modalities and adjustments to existing treatments have been proposed to make treatment as little toxic as possible. Good short-term results have been reported from the anti-CD20 antibody rituximab [11] as monotherapy, but relapses occur frequently [20–22]. Possibly, a combination of rituximab and chemotherapy will result in better long-term disease control. Very low-dose involved-field radiotherapy, with a dose of 2 × 2 Gy as used in indolent non-Hodgkin lymphoma, can be an effective alternative treatment in frail patients or in those with contraindications for more intense regimens [23]. A recent German study showed similar results for combined modality treatment, extended-field radiotherapy, and involved-field radiotherapy in patients with stage IA NLPHL and concluded that involved-field radiotherapy should be considered as standard treatment because it carries the lowest risk of toxicity [22].

Seven patients (9.6 %) in our cohort had a relapse, which is comparable to the 10 % relapse rate in a recent study from Farrell et al. [9]. In the study with the highest numbers of patients with a watch-and-wait strategy, 40 % relapsed within a median follow-up of 9.5 years [18]. In the cohort where no patients were on a watch-and-wait strategy, 23 % relapsed within a median follow-up of 11.3 years [24]. Histologic transformation during the follow-up period occurred in six of our patients (8.2 %), which is comparable to percentages given in literature that range from 2.7 to 13 % [9, 15]. Transformation to aggressive lymphoma can increase up to 30 % at 20 years after initial diagnosis [15].

Five patients (6.8 %) died during follow-up, three due to NLPHL, and two because of other causes. In the study of Farrell, the overall survival was 100 % with a median follow-up of 53 months [9]; the reported death rates in other recent studies varied from 8.5 to 16.8 % [1].

In conclusion, the results from our study corroborate the results of other recent studies and may serve to develop international joint strategies to optimize treatment for patients with NLPHL. Only well-designed randomized intergroup trials can provide results for developing the best treatment in a good balance between efficacy and toxicity. Since we still found a discordance rate of 8.8 %, we strongly recommend central review by an expert hematopathologist in the initial diagnosis as reported earlier in a cohort of patients with Hodgkin lymphoma in the Southeast of the Netherlands [25].
